# The Basic Surgical Skills Course in Sub-Saharan Africa: An Observational Study of Effectiveness

**DOI:** 10.1007/s00268-017-4274-2

**Published:** 2017-10-20

**Authors:** Stuart J. Fergusson, David M. Sedgwick, Georges Ntakiyiruta, Faustin Ntirenganya

**Affiliations:** 1 University of Glasgow, University Avenue, Glasgow, G12 8QQ UK; 2 Fort William, Lochaber UK; 3 Ejo Heza Surgical Centre, KN 25, St 9, Kiyovu Sector, Nyarugenge District, Ishema Village, Kigali, Rwanda; 4 University Teaching Hospital of Kigali, KN 4 Ave, Kigali, Rwanda

## Abstract

**Background:**

The Basic Surgical Skills (BSS) course is a common component of postgraduate surgical training programmes in sub-Saharan Africa, but was originally designed in a UK context, and its efficacy and relevance have not been formally assessed in Africa.

**Methods:**

An observational study was carried out during a BSS course delivered to early-stage surgical trainees from Rwanda and the Democratic Republic of the Congo. Technical skill in a basic wound closure task was assessed in a formal Objective Structured Assessment of Technical Skills (OSAT) before and after course completion. Participants completed a pre-course questionnaire documenting existing surgical experience and self-perceived confidence levels in surgical skills which were to be taught during the course. Participants repeated confidence ratings and completed course evaluation following course delivery.

**Results:**

A cohort of 17 participants had completed a pre-course median of 150 Caesarean sections as primary operator. Performance on the OSAT improved from a mean of 10.5/17 pre-course to 14.2/17 post-course (mean of paired differences 3.7, *p* < 0.001). Improvements were seen in 15/17 components of wound closure. Pre-course, only 47% of candidates were forming hand-tied knots correctly and 38% were appropriately crossing hands with each throw, improving to 88 and 76%, respectively, following the course (*p* = 0.01 for both components). Confidence levels improved significantly in all technical skills taught, and the course was assessed as highly relevant by trainees.

**Conclusion:**

The Basic Surgical Skills course is effective in improving the basic surgical technique of surgical trainees from sub-Saharan Africa and their confidence in key technical skills.

## Introduction

Recent advocacy efforts have starkly highlighted the crucial importance of improving surgical provision in the world’s poorest countries and have emphasised to healthcare decision-makers that investing in surgical services in low- and middle-income countries is “affordable, saves lives and promotes economic growth” [1]. The dearth of surgical provision in sub-Saharan Africa, in particular, is well known [2, 3]. African postgraduate surgical training programmes have significantly developed over recent years [4], and one of the most widespread taught components of early surgical training is the Basic Surgical Skills (BSS) course.

The BSS course was originally developed in 1994 by all of the Royal College of Surgeons in the UK and Ireland, with the philosophy of teaching “one safe way” of performing key technical tasks required of surgeons [5]. This was the result of an influential report into UK postgraduate specialist training published in 1993 (“The Calman report”) which recommended greater standardisation of the teaching of basic surgical skills [6]. The BSS course became mandatory for UK basic surgical trainees in 1996, with the course subsequently spreading to use in Australia, Africa, Southeast Asia and many European countries [7]. It was comprehensively revised in 2012 to improve trainee assessment and include an Objective Structured Assessment of Technical Skills (OSAT) [8]. The BSS course is conducted over an intense 2-day period, and the curriculum progresses from an initial reinforcement of sterility, basic instrument handling and knot tying, to an application of these principles in minor skin procedures, bowel and vascular anastomosis, and tendon repair. In lower-resource settings, the introductory sessions on laparoscopic surgery are generally replaced with teaching on cricothyroidotomy, suprapubic catheter insertion, chest drain insertion and application of a plaster cast for Colles’ fracture. The BSS course combines demonstrations, individual tutoring and extensive personal practical exercises to achieve its aims, with the use of high-quality synthetic and animal simulations of human tissues.

While the BSS course has proved popular and internationally successful [5], it was designed in a UK context. While there is good evidence of this course’s popularity in a sub-Saharan African context [9–11], the experience and educational needs of early-stage African surgical trainees, from resource-challenged environments, may be different from their Western counterparts [12], for whom the course was originally designed.

This study therefore aimed to describe the technical skills and experience of African surgical trainees at the beginning of their postgraduate training, assess whether the Basic Surgical Skills course improves trainee technical performance and confidence and assess trainees’ perception of the course.

## Materials and methods

### Context

An observational study was conducted within a Basic Surgical Skills course conducted at the University Teaching Hospital of Kigali, Rwanda, in October 2016. Course participants were at the early stages of postgraduate surgical training programmes and were recruited from both Rwanda and the Democratic Republic of the Congo (DRC). The need for surgical capacity building is acute in Rwanda and DRC, where there are surgeon densities of 0.4 and 0.1 per 100,000 population, respectively [13]. In Rwanda, most surgeons work in the capital, Kigali, covering only 10% of the population [14].

The course was convened by an experienced consultant surgeon tutor from the Royal College of Surgeons of Edinburgh (DMS) and was largely delivered by a Rwandan faculty of consultant surgeons, who had all completed a “training the trainers” preparation course for BSS instructors.

### Protocol

Participants were recruited from this BSS course cohort, and after giving written informed consent, they completed an initial questionnaire, which collated basic demographics, baseline operative experience in selected procedures and confidence levels in a variety of basic surgical skills, using a 5-point Likert scale. These basic surgical skills were chosen to match BSS course contents and the 2016 syllabus of the College of Surgeons of East and Central Southern Africa (COSECSA) for their membership examination (Sect. 7.1). A 17-point Objective Structured Assessment of Technical Skills (OSAT) was performed prior to formal course commencement, under examination conditions. This OSAT assessed performance on a basic wound closure task involving hand tying of sutures and was examined by faculty who all had previous experience of administering this test. At this stage, participants did not receive any feedback on their performance. Following delivery of course content, participants repeated an identical OSAT and repeated confidence ratings using the same questions. Participants also completed a course evaluation tool following course completion, where overall impressions and clarity, relevance and delivery of individual course components were assessed using 5-point scales.

### Analysis

OSAT scores were compared before and after course completion by using a paired *t* test, and the proportion of correctly completed components of the test (e.g. suture placement, correct knot configuration) were compared before and after the course using a *χ* ^2^ test. Confidence levels were assessed by first creating a binary outcome of confident/unconfident, through compression of the 5-point categorisation. Confidence was then compared pre- and post-BSS course using a *χ* ^2^ test.

### Power calculation

A power calculation had been carried out to determine the required sample size. In the 2015 Rwandan Basic Surgical Skills course cohort, mean score on the OSAT test of technical ability (following the course) was 14.2/17, with standard deviation 1.59. A difference of 3/17 in test scores was thought to be of educational significance. It was determined using a matched pairs t test model that a total sample size of 6 would be sufficient to demonstrate this difference in scores with α 0.05 and power of 0.95 (G*Power version 3.1.9.2 [15]).

## Results

### Background characteristics

A total of 17 surgical trainees attended the BSS course, and all chose to participate in the study. Table 1 describes cohort characteristics, and Table 2 describes the pre-course operative experience of the cohort. Although 71% of participants had worked as a doctor for less than 3 years, they attended the course with significant surgical experience, particularly in Caesarean section (median procedure count 150).

**Table 1:** Cohort characteristics

		Number of participants (% within category)
Country of graduation and current workplace	Rwanda	13 (76%)
	Democratic Republic of the Congo	4 (24%)
Completed whole years since graduation	1 year	6 (35%)
	2–3 years	6 (35%)
	≥ 4 years	5 (29%)
Gender	Male	15 (88%)
	Female	2 (12%)
Desired future specialty	General surgery	12 (71%)
	Neurosurgery	2 (12%)
	Orthopaedics	1 (6%)
	Urology	2 (12%)

**Table 2:** Previous operative experience

	Number of procedures performed as the main operator (median, inter-quartile range)	Number of procedures performed as an assistant (median, inter-quartile range)
Caesarean section	150, 60–250	100, 40–170
Inguinal hernia repair	0, 0–4	25, 10–30
Appendicectomy	0, 0–1	5, 1–15
Emergency laparotomy	0, 0–3	10, 7–20
Wound debridement	15, 10–30	20, 10–35
Chest drain insertion	1, 0–2	4, 2–10
Suprapubic catheter insertion	1, 0–5	3, 1–6
Closed reduction/POP for fracture	10, 4–58	18, 10–50
Open reduction/fixation of fracture	0, 0–2	20, 10–30

### Technical performance

Participants demonstrated a significant improvement in OSAT performance following the course. The mean pre-course OSAT score (out of 17) was 10.5, with standard deviation (SD) 2.1. Following the course, mean OSAT score improved to 14.2 (SD 1.4). The mean of paired pre-/post-OSAT score difference was 3.7 (SD 2.0), with *p* value < 0.001 (paired *t* test), 95% confidence interval 2.7–4.7.

Table 3 illustrates performance in individual components of the OSAT before and after the BSS course. Overall cohort performance improved in 15 of the 17 assessed components of the basic suturing task, the difference in which reached statistical significance in 7 components. Although participants began the course with significant procedural experience, before the BSS course, they were deficient in several aspects of basic suturing technique. For example, only 41% of participants were appropriately mounting and orientating the needle in the jaws of the needle holder, 41% were taking the needle in an appropriate trajectory through the tissues, 47% were appropriately forming each throw of the knot, and only 38% were appropriately crossing hands with each throw. Improvement in each of these key aspects of basic surgical skill reached statistical significance following the BSS course. Table 3 also highlights pre-existent deficiencies in key aspects of safe surgical practice—such as avoidance of needle handling and safe put-down/disposal of the needle (only achieved by 41 and 35% of participants, respectively, before the BSS course). Reassessment following the course showed improvement in these two areas (*p* values < 0.01 and 0.09, respectively).

**Table 3:** Itemised technical performance in OSAT components: before and after course (number of participants performing correctly, %)

	Before course	After course	*p* value (*χ* ^2^ test)
Safe removal of suture from packet	15 (88%)	17 (100%)	0.14
Safe mounting on needle holder	10 (59%)	17 (100%)	<0.01
Appropriate mounting and orientation of needle in jaws	7 (41%)	16 (94%)	<0.001
Counter traction on tissue by forceps	10 (59%)	14 (82%)	0.13
Appropriate suture bite size	10 (63%)*	15 (88%)	0.03
Appropriate trajectory of needle through tissues	7 (41%)	14 (82%)	0.01
Appropriate formation of each throw of knot	8 (47%)	15 (88%)	0.01
Appropriate crossing of hands with each throw	6 (38%)*	13 (76%)	0.01
Appropriate number of throws for suture material used	12 (75%)*	13 (76%)	0.67
Correct suture tension: not pulled too tight	9 (53%)	12 (71%)	0.29
Correct suture tension: not left too loose	13 (76%)	14 (82%)	0.67
Correct cut/length of suture	14 (82%)	13 (76%)	0.67
Correct distance between sutures	17 (100%)	11 (65%)	<0.01
Avoided handling needle	7 (41%)	14 (88%)*	<0.01
Safe put-down/disposal of needle	6 (35%)	11 (65%)	0.09
Correct stitch removal technique	10 (59%)	14 (82%)	0.13
Completed task with suture length provided	16 (94%)	17 (100%)	0.31

* These percentages calculated from a denominator of 16 (one rating missing on these items)

### Personal confidence levels

The BSS course resulted in a significant improvement in confidence levels across each of the eight surgical skills where confidence was assessed (Table 4), with almost all participants achieving confidence by the course conclusion. Pre-course confidence levels were low in most of the basic surgical tasks which are taught in BSS.

**Table 4:** Confidence levels: before and after course (number of confident/very confident participants, %)

	Before course	After course	*p* value (*χ* ^2^ test)
Hand tying a surgical knot	4 (24%)	17 (100%)	<0.001
Proper surgical instrument handling/suturing	11 (65%)	17 (100%)	<0.01
Principles for safe use of diathermy	5 (29%)	16 (94%)	<0.001
Excision of a simple skin lesion (e.g. cyst)	11 (65%)	17 (100%)	<0.01
Opening/closing an abdomen for laparotomy	8 (47%)	16 (94%)	<0.01
Bowel anastomosis	3 (18%)	17 (100%)	<0.001
Chest drain insertion	4 (24%)	17 (100%)	<0.001
Suprapubic catheter insertion	7 (41%)	17 (100%)	<0.001

### Overall course evaluation

Formal course evaluation was very positive. Of the 15 participants who provided overall ratings of the course, 11 rated it “excellent” (73%) and 4 rated it “good” (27%). All these participants stated that the course had met their expectations and that they would recommend it to a friend or colleague. In an evaluation of individual course components (Table 5), the mean ratings of clarity, relevance and delivery ranged from 4.3 to 5.0 (on a 5-point Likert scale where 5 was “very good”).

**Table 5:** Overall evaluation of course components (mean scores) (1 = very poor, 2 = poor, 3 = adequate, 4 = good, 5 = very good)

Sessions	Clarity of information	Relevance of session	Delivery (materials/style)
Day 1			
Introduction	4.6	4.8	4.8
Gowning and gloving	4.9	4.8	4.8
Handling instruments	4.8	4.9	4.9
Knots (1): reef knot, instrument-tied	4.9	4.9	4.9
Knots (2): surgeons knot, tie at depth	4.9	4.9	4.9
Suturing: needle manipulation and driving, interrupted, sub-cuticular, vertical mattress	4.9	4.9	4.8
Skin lesions and local anaesthetic techniques—skin lesions, sebaceous cyst	4.7	4.9	4.8
Fine tissue handling (1): tendon repair, assisted	4.9	4.9	4.9
Wound management—abscess drainage, debriding traumatic wound	5.0	4.9	4.9
Diathermy	4.3	4.5	4.5
Day 2			
Skills consolidation/OSAT	4.7	4.7	4.7
Abdominal incision and closure	4.8	4.8	4.6
Haemostasis—ligation in continuity, pedicle ligation, transfixion	4.8	4.9	4.9
End-to-end bowel anastomosis—interrupted, instrument-tied knots	4.9	4.9	4.8
Fine tissue handling (2): vein patch exercise, assisted	4.6	4.6	4.4
Chest drain insertion	4.9	4.9	4.8
Cricothyroidotomy	4.6	4.6	4.4
Insertion of suprapubic catheter	4.9	4.9	4.8
Application of Colles’ plaster cast	4.8	4.9	4.9

## Discussion

### Summary

This study objectively demonstrates the continuing utility of the Basic Surgical Skills course in improving the technical performance of African surgical trainees in a basic suturing task and in improving their confidence levels across a range of surgical skills. This study highlights that although surgical trainees from a resource-challenged African environment will begin their surgical training with substantial operating experience, they still lack ability in key technical skills and lack confidence in some basic surgical tasks. These trainees rate all aspects of the BSS course highly, and it appears to deliver on its objectives.

### Study strengths

While there are existing reports of the acceptability and popularity of the BSS course in sub-Saharan Africa [9–11], this is the first study we are aware of which provides objective evidence for its effectiveness in improving African surgical trainee performance in technical skills and improving their confidence in key basic surgical tasks. A previous study of BSS courses in Nigeria showed similarly low levels of pre-course confidence in the taught skills, but did not quantify whether improvement occurred following course delivery [9]. This study was performed in a high-quality educational environment, with several very experienced faculty members and an instructor-to-student ratio of approximately 1:2. Some observational evidence shows that the instructor-to-student ratio required to optimise suturing skills learning is 1:4 [16], a ratio which was comfortably exceeded in this course. Immediately prior to the study, all new BSS instructors were taught the four-stage method of clinical skill teaching, which is proven to result in students performing technical tasks faster and better [17]. A very similar technical task was shown in another context to clearly differentiate grade of surgeon, suggesting that this assessment is a robust measure of technical ability [18].

### Study limitations

There is a theoretical possibility that the improvement in technical skill observed in this study was simply a “testing effect” [19]. However, mitigating against this possibility is the fact that no feedback was offered to participants following the pre-course OSAT test. Furthermore, when the BSS was performed a year ago in Kigali under the same conditions with the same number of candidates, the mean post-course OSAT mark achieved was an identical 14.2/17, suggesting that this study cohort did not derive a meaningful advantage from earlier completion of an identical OSAT. In terms of generalisability, although the Basic Surgical Skills course content and delivery is standardised, instructor–student dynamics and instructor-to-student ratios will vary. The experience in a single course may not be replicable in every sub-Saharan African context. We also recognise that our assessment tool, the OSAT, has not undergone formal validation although it has been very widely used. Lastly, the OSAT includes an assessment of hand tying which could be criticised as less applicable to the practice of a surgeon in a low-income setting, who may preferentially instrument-tie in order to save suture. However, we maintain that hand tying remains a key basic surgical skill which is an appropriate area for assessment.

### Implications

The results of this study underline the continuing relevance and effectiveness of the Basic Surgical Skills course in sub-Saharan Africa and support the current requirement for surgical trainees to have completed a BSS course before being awarded COSECSA’s membership diploma, MCS (ECSA). While there is no published evidence of the overall impact of the BSS course on future practice, there are good grounds for believing that effective teaching of technical skills will result in better clinical care. A comparative study of obstetric trainees showed a significant increase in knot strength following a targeted knot tying workshop and a reduction in the proportion of unsafe, weak knots [20].

In African healthcare systems where so much surgical practice is conducted by non-surgeons, advocates have already argued that basic surgical skills teaching should be made more widely available [21]. The extent of Caesarean section experience amongst our cohort and their poor results on some components of the pre-course OSAT highlight that a large volume of theatre work in Rwanda is being undertaken by junior doctors who may have shortcomings in basic surgical techniques. In a healthcare system which relies on junior staff to carry out essential surgical procedures such as Caesarean section, our findings suggest that formal surgical skills training courses such as the BSS may be more appropriately targeted at final year medical students or first year interns, rather than early-stage surgical trainees.

African national surgical training programmes now increasingly have the capacity to keep training in-country, which is seen as key to retaining staff and effectively building capacity [22, 23]. The pattern of outsourcing services in surgical “blitzes” encourages systems of dependency and may result in poorer outcomes than routine care [24]. This study’s findings place an obligation on African training programmes to make appropriate strategic and financial plans for the sustainable continuation of the Basic Surgical Skills training course.
